# Synthesis, characterization and *n*-hexane hydroisomerization performances of Pt supported on alkali treated ZSM-22 and ZSM-48

**DOI:** 10.1039/c8ra04858d

**Published:** 2018-08-14

**Authors:** Haoran Li, Chenglian Liu, Yan Wang, Jiajun Zheng, Binbin Fan, Ruifeng Li

**Affiliations:** College of Chemistry and Chemical Engineering, Taiyuan University of Technology Taiyuan 030024 China wangyang@tyut.edu.cn rfli@tyut.edu.cn

## Abstract

ZSM-48 and ZSM-22 zeolites with similar Si/Al molar ratio have been treated with alkali to modify the pore structures and acidity, and alkali treated ZSM-22 and ZSM-48 samples have been characterized by X-ray Diffraction (XRD), Scanning Electron Microscopy (SEM), Transmission Electron Microscopy (TEM), N_2_ adsorption/desorption, Nuclear Magnetic Resonance (NMR), NH_3_-Temperature Programmed Desorption (NH_3_-TPD) and Pyridine-Fourier Transform Infrared Spectroscopy (Py-FTIR). Characterization results indicate that NaOH treatment could improve the mesoporous structure for both ZSM-22 and ZSM-48. NaOH treatment modifies the acidity of ZSM-22 and ZSM-48 diversely. The *n*-hexane hydroisomerization performances of Pt supported protonic form ZSM-22 and ZSM-48 (Pt/HZSM-22 and Pt/HZSM-48) bifunctional catalysts have been evaluated in a fixed bed reactor. Catalytic results indicate that catalytic activity and selectivity depend on both pore structure and acidity of zeolites. In comparison of Pt/HZSM-22 and Pt/HZSM-48, Pt/HZSM-22 shows better *n*-hexane hydroisomerization performance at relatively low temperature (<300 °C), meanwhile, at relatively high temperature (>300 °C) Pt/HZSM-48 exhibits better catalytic performance. Moreover, alkali treated Pt/HZSM-48 could produce more di-branched isomer compared with alkali treated Pt/HZSM-22.

## Abstract 2

ZSM-48 and ZSM-22 zeolites with similar Si/Al molar ratio have been treated with alkali to modify the pore structures and acidity, and they have been characterized by XRD, SEM, TEM, N_2_ adsorption/desorption, NMR, NH_3_-TPD and Py-FTIR.

## Graphical abstract

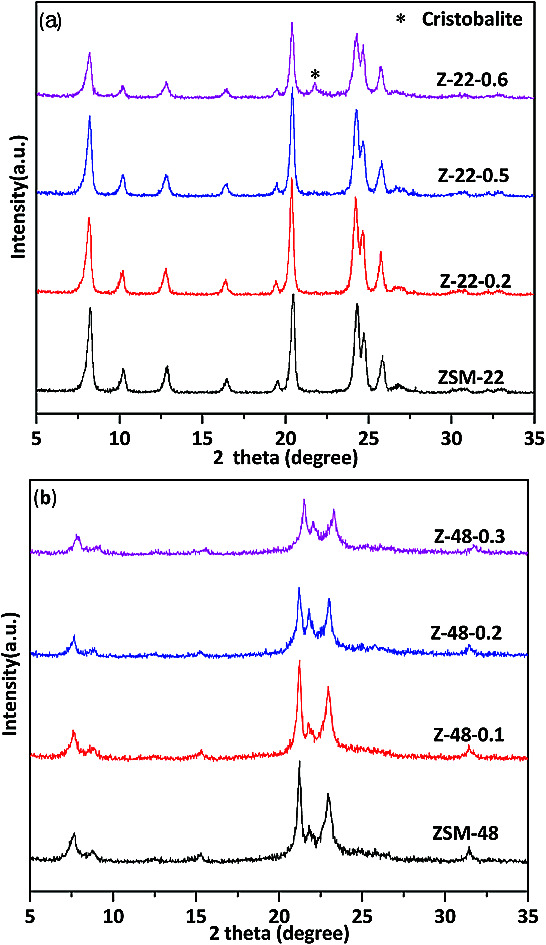

## 1. Introduction

With the rapid development of the economy and automobile industry, the requirements for high quality oil products and clean fuel have spontaneously grown. The quality of oil can be improved by modifying raw materials' molecular structure. Hydroisomerization of *n*-alkane is a reaction that can both decrease pour point of oil, and maintain high coefficient of viscosity and yield. Hydroisomerization technology was one of the most important improvements in the oil refinery industry and the core of the hydroisomerization technology is the development of highly efficient catalysts.^1^ C5–C6 alkane hydroisomerization, also known as light naphtha isomerization, aims to increase the octane number of light naphtha, but also contributes to benzene management and cycloalkane ring opening in refineries.

Hydroisomerization catalysts are usually bifunctional catalysts, including two active components of supports (acid sites) and metals (hydrogenation/dehydrogenation sites), where supports provide both acid sites for isomerization and suitable pore structures for shape selectivity, and metals provides hydrogenation/dehydrogenation sites. Noble metals such as Pt are usually chosen as hydrogenation components, due to their excellent ability of avoiding coke formation.^2,3^ Meanwhile, zeolites such as beta, ZSM-5, ZSM-22, ZSM-23, and ZSM-48 *etc.* are often chosen as acid sites components. ZSM-48 and ZSM-22 are potential candidates for hydroisomerization reaction due to the one-dimensional 10-membered ring channel structures and suitable acidity.^4–9^ However, common used ZSM-48 with relatively high Si/Al molar ratio greatly hindered its application for isomerization due to its low acidity.^10^ Thus, in this paper ZSM-48 with relatively low Si/Al molar ratio (Si/Al = 40) have been successfully synthesized, and ZSM-48 have been further treated by alkali to modify its pore structure and acidity. Meanwhile, commercial ZSM-22 (Si/Al = 37) with similar Si/Al as as-synthesized ZSM-48 was treated by alkali, and both alkali treated ZSM-22 and ZSM-48 have been characterized by XRD, SEM, TEM, NMR, N_2_ adsorption/desorption, NH_3_-TPD and Py-FTIR to investigate their structure and acidity. The correlation between pore structure and acidity with the hydroisomerization performances of *n*-hexane on Pt supported HZSM-22 and HZSM-48 have also been investigated.

## 2. Experiments

### 2.1 Materials

Silica gel with 40 wt% of SiO_2_ was purchased from Qingdao Ocean Chemical Co., Ltd. Sodium aluminates (NaAlO_2_) with Al_2_O_3_ weight ratio more than 41% was provide by Sinopharm Chemical Reagent Co., Ltd. NaOH, ammonium chloride and *n*-hexane (analytical reagents, AR) were purchased from Tianjin Kemiou Chemical Reagent Co., Ltd. Hexamethonium bromide (RBr, 98 wt%) was provided by J&K Chemical Ltd. Analytical reagent H_2_PtCl_6_·6H_2_O was purchased from Shanghai Aladdin Bio-Chem Technology Co., LTD. All reagents were used as received without further purification.

### 2.2 Catalyst preparation

#### 2.2.1 ZSM-22 modification

Alkali treatments of ZSM-22 were conducted as follows: commercial ZSM-22 with Si/Al = 37 was treated by different concentration of NaOH solution (0.2 mol L^−1^, 0.5 mol L^−1^ and 0.6 mol L^−1^) by stirring at 80 °C for 2 h, the weight ratio of solid to liquid was fixed to 1 : 30. ZSM-22 treated with different NaOH concentrations (0.2 mol L^−1^, 0.5 mol L^−1^ and 0.6 mol L^−1^) were denoted as Z-22-0.2, Z-22-0.5 and Z-22-0.6, respectively. Alkali treated ZSM-22 powders were ion-exchanged 3 times by 1 mol L^−1^ NH_4_Cl solution containing 0.1 mol L^−1^ HCl at 80 °C for 2 h under agitation, the weight ratio of solid to liquid was also fixed to 1 : 30. NH^4+^-exchanged ZSM-22 powders were calcined at 550 °C in air for 6 h to obtain protonic form HZSM-22.

#### 2.2.2 ZSM-48 synthesis and modification

Precursor gel was synthesized by dropwise adding silica gel to a solution of NaAlO_2_, NaOH and RBr under stirring with a molar composition of 1SiO_2_:0.01Al_2_O_3_:0.018RBr:0.09NaOH:18H_2_O, then transferred into a stainless steel autoclave and subjected to hydrothermal treatment at 180 °C for 17 h in an oven under static condition.

Alkali treatments of ZSM-48 were conducted as follows: as-synthesized ZSM-48 powders were mixed with different concentration of NaOH solution (0.1 mol L^−1^, 0.2 mol L^−1^ and 0.3 mol L^−1^) under agitation at 80 °C for 1 h. The weight ratio of ZSM-48 to NaOH solution was also fixed to 1 : 15, and ZSM-48 zeolites treated with 0.1 mol L^−1^, 0.2 mol L^−1^ and 0.3 mol L^−1^ NaOH were denoted as Z-48-0.1, Z-48-0.2, and Z-48-0.3, respectively. Alkali treated zeolites were converted into protonic form (HZSM-48) by ion exchange 3 times with 1 mol L^−1^ NH_4_Cl solution at 80 °C for 1 h, followed by calcination at 550 °C in air for 4 h.

To obtain catalysts for hydroisomerization, protonic formed samples were impregnated into a solution of H_2_PtCl_6_·6H_2_O, and then the suspension was slowly evaporated at 80 °C. The obtained samples were then calcined at 550 °C for 6 h. Pt loaded on HZSM-22 or HZSM-48 was 0.5wt%. The obtained catalysts were denoted as Pt/HZSM-22, Pt/HZ-22-0.2, Pt/HZ-22-0.5, Pt/HZ-22-0.6, Pt/HZSM-48, Pt/HZ-48-0.1, Pt/HZ-48-0.2 and Pt/HZ-48-0.3, respectively.

### 2.3 Characterizations

XRD patterns were recorded on a SHIMADZU XRD-6000 X-ray diffractometer at 40 kV and 80 mA, using Cu Kα radiation. SEM and TEM images were recorded by using a HITACHI S-4800 scanning electron microscope and a JEOL JSM-6700F transmission electron microscope, respectively. Surface areas, pore volume and pore size distribution of samples were determined by analyzing N_2_ adsorption/desorption isotherms on a Quantachrome QUADRASORB SI instrument at −196 °C. Surface area was calculated by BET (Brunauer–Emmett–Teller) method. Microporous volumes were calculated by using *t*-plot method. Pore size distribution and mesoporous volumes were calculated from the adsorption branch of the N_2_ adsorption/desorption isotherm according to DFT model. Temperature-programmed desorption of hydrogen (H_2_-TPD) experiment was carried out in a Micromeritics Autochem II 2920 instrument. The sample was packed into a reactor with quartz tubing, and was treated at 500 °C for 2 h under a flow of hydrogen (flow rate of 30 mL min^−1^). After cooled down to room temperature, the catalyst was purged in flow of argon (30 mL min^−1^) at for 1 h. H_2_-TPD experiment was performed by sample heating at a rate of 10 °C min^−1^ from room temperature to 600 °C under an argon flow (30 mL min^−1^). The amount of desorbed hydrogen was measured by a TCD detector.

^29^Si and ^27^Al MAS NMR experiments were conducted on a Bruker Avance III spectrometer. Pyridine adsorbed FTIR spectra (Py-FTIR) were recorded on a SHIMADZU 8400 Fourier Transform Infrared spectrometer, using a self-supporting wafer in an *in situ* cell. The wafers of 12 mg samples were first pre-treated at 450 °C for 1 h in vacuum of 10^−3^ Pa, and then cool down to room temperature, followed by pyridine adsorption. Py-FTIR spectra were recorded after degassing for 20 minutes at 150 °C and 300 °C in vacuum, respectively. The spectra of pyridine adsorption were obtained by absorbance-subtraction of the sample background spectrum. NH_3_-TPD experiments were performed on a TP-5076 instrument (XIANQUAN, China) equipped with a thermal conductivity detector (TCD). 20 mg of catalyst was loaded in a quartz tube, and then sample was pre-treated at 550 °C for 1 h in He flow. After cooling down to 120 °C, the sample was saturated with 10 wt% NH_3_/He gas mixture (50 mL min^−1^) for 0.5 h, followed by He purging for 0.5 h. Then, the temperature was increased at 10 °C min^−1^ in 30 mL min^−1^ He flow to 700 °C, and the signals of NH_3_ desorption were recorded.

### 2.4 Catalytic performance

Hydroisomerization of *n*-hexane was carried out in a continuous flow fix-bed reactor. Catalytic test was performed at atmospheric pressure, hydrogen to hydrocarbon volume ratio of 1000, and liquid hourly space velocity (WHSV) of 1.0 h^−1^. Catalyst was first pre-treated at 400 °C for 2 h under H_2_ atmosphere. After reduction procedure, catalyst was cooled down to 280 °C, followed by *n*-hexane pumped into reactor by a microscale pump. Hydroisomerization reaction was carried out in a temperature range of 280–360 °C. For catalytic test of each catalyst, five temperature points were sampled at an interval of 2 h for each sampling period, in which temperature was kept constant. The products were analyzed by an online gas chromatograph (PANNA A91, China). After each sampling period, reaction temperature was increased to higher temperature for next catalytic test.

## 3. Results and discussion

### 3.1 Physicochemical properties of catalysts

**Fig. 1:**
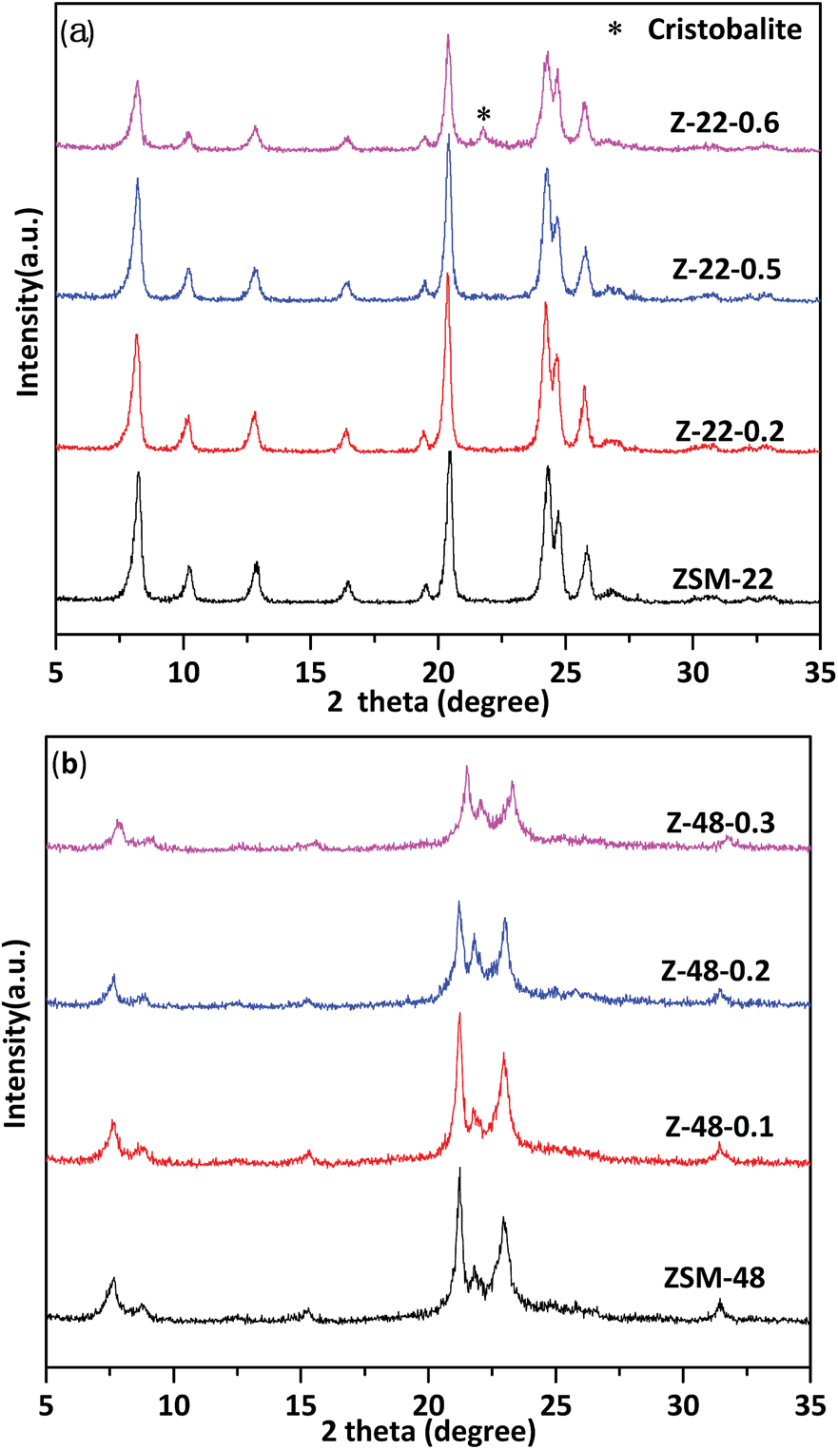
XRD patterns of ZSM-22 (a) and ZSM-48 (b) treated with different NaOH concentrations.

XRD patterns of untreated ZSM-22 and ZSM-22 treated by 0.2 mol L^−1^ (Z-22-0.2), 0.5 mol L^−1^ (Z-22-0.5), and 0.6 mol L^−1^ (Z-22-0.6), respectively, are shown in Fig. 1a. The identical diffraction peaks of TON-type zeolites are presented at 8.3°, 20.5°, 24.3°, 24.8° and 25.9° on all samples, indicating the samples retain ZSM-22 topological structure after treated by NaOH solution. However, the relative crystallinity of ZSM-22 decreases along with the NaOH solution concentration increasing (see Table 1). Along with NaOH concentration increasing to 0.6 mol L^−1^, the relative crystallinity of ZSM-22 decrease to 65% of its original level. Meanwhile, clear impurity diffraction peak of cristobalite can be visualized on sample Z-22-0.6. XRD patterns of untreated ZSM-48 and ZSM-48 treated NaOH by 0.1 mol L^−1^ (Z-48-0.1), 0.2 mol L^−1^ (Z-48-0.2), and 0.3 mol L^−1^ (Z-48-0.3), respectively, are shown in Fig. 1b. As illustrated in Fig. 1b, the characteristic diffraction peaks of ZSM-48 at 7.6°, 15.0°, 21.0°, 22.7° and 31.2° are presented. This result suggests pure ZSM-48 zeolite is successfully synthesized and the topological structure retains after treated at NaOH concentration range 0.1–0.3 mol L^−1^. Meanwhile, as ZSM-22 the relative crystallinity of ZSM-48 decreased after NaOH treatments (see Table 1), especially when NaOH treatment concentration is 0.3 mol L^−1^ the relative crystallinity decrease to 74% of its original level. Moreover, no impurity diffraction peak can be visualized on all samples.

**Table 1:** Structure parameters of ZSM-22 and ZSM-48 samples

Sample	*S*_BET_ (m^2^ g^−1^)	*V*_mic_ (cm^3^ g^−1^)	*S*_mic_ (m^2^ g^−1^)	*S*_ext_ (m^2^ g^−1^)	*V*_total_ (cm^3^ g^−1^)	*V*_meso_ (cm^3^ g^−1^)	Relative crystallinity
ZSM-22	88	0.017	41	47	0.23	0.21	100%
Z-22-0.2	88	0.015	32	56	0.28	0.26	96%
Z-22-0.5	121	0.017	40	81	0.45	0.43	92%
ZSM-48	222	0.066	162	60	0.07	0.00	100%
Z-48-0.1	257	0.055	132	125	0.14	0.09	94%
Z-48-0.2	285	0.059	141	144	0.26	0.21	85%
Z-48-0.3	284	0.06	135	149	0.26	0.20	74%

**Fig. 2:**
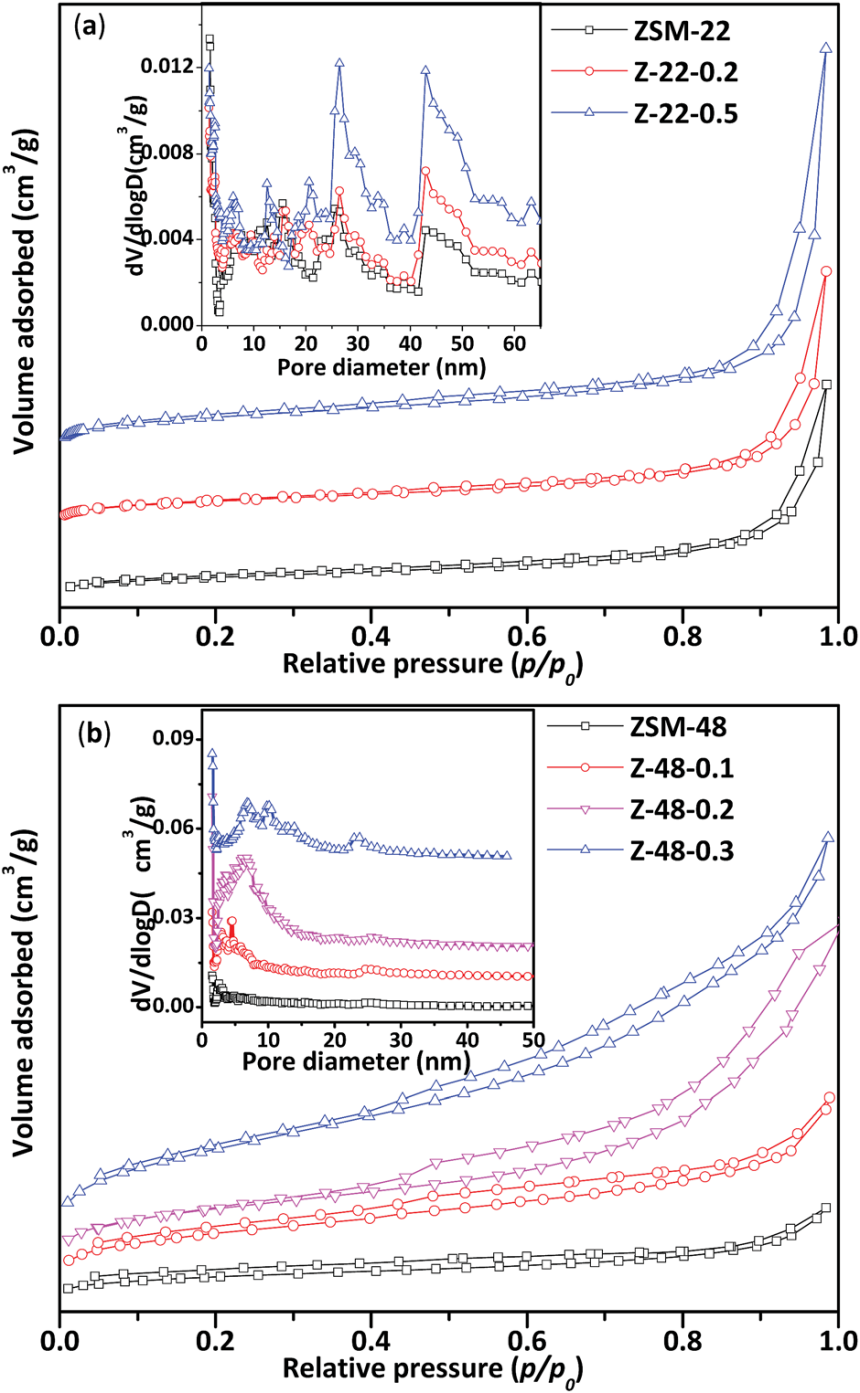
N_2_ adsorption/desorption isotherms of ZSM-22 (a) and ZSM-48 (b) and corresponding pore size distributions of ZSM-22 and ZSM-48.

N_2_ adsorption/desorption isotherms (a and b) and corresponding pore size distributions (insets) of ZSM-22 and ZSM-48 samples are displayed in Fig. 2. Structure parameters obtained from N_2_ adsorption/desorption isotherms are listed in Table 1. All ZSM-22 samples obtained mesopores in both untreated and NaOH treated ZSM-22 (see Table 1). After alkali treatment, more mesoporous structures are introduced in ZSM-22. However, the size of ZSM-22 original mesopores has not been enlarged obviously. It can be seen that pore size distribution of ZSM-22 (Fig. 2a inset) is wide with the range all over 5–50 nm. With NaOH concentration increasing, mesoporous volume (*V*_meso_) increases from 0.21 to 0.43 cm^3^ g^−1^, BET surface area (*S*_BET_) increases from 88 to 121 m^2^ g^−1^, and external specific surface area (*S*_ext_) increases from 47 to 81 m^2^ g^−1^. This result indicated that NaOH treatment can produce more mesoporous structures, at the same time microspores of ZSM-22 zeolite in the alkali treatment process have been largely retained.

It can be seen that the N_2_ adsorption/desorption isotherms of untreated ZSM-48 is a type “I” isotherm, indicating that the synthesized ZSM-48 is a typical microporous structure material. ZSM-48 sample after NaOH treatment begin to appear an obvious hysteresis loop at *p*/*p*_0_ = 0.4, indicating the mesoporous structure is introduced into ZSM-48. It can be seen that the pore size distribution of ZSM-48 (Fig. 2b inset) with different NaOH concentration treated is ranged from 5 to 15 nm. Along with NaOH concentration increasing, larger mesoporous structures have been created. With NaOH concentration increasing, more mesoporous appear and the original microporous structure is largely retained.

N_2_ adsorption/desorption results indicate that the pore structures of ZSM-22 and ZSM-48 both have been obviously modified by NaOH treatment. With NaOH concentration increasing, BET surface area, external surface area and mesoporous volume have been improved. This result is in agreement with literature reports.^11,12^ However, combined with XRD results that optimal NaOH treatment concentration of ZSM-22 and ZSM-48 are different, NaOH concentration should be lower than 0.5 mol L^−1^ for ZSM-22, and for the ZSM-48 it should be below 0.3 mol L^−1^.

**Fig. 3:**
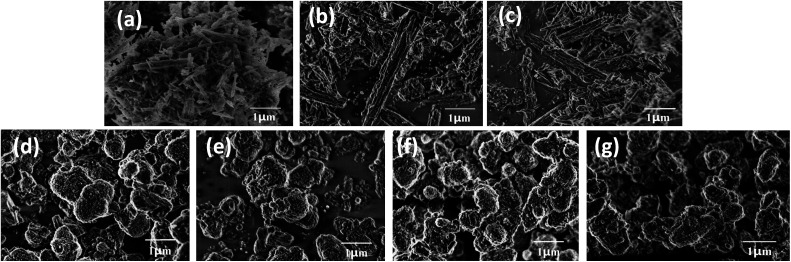
SEM images of ZSM-22 (a), Z-22-0.2 (b), Z-22-0.5 (c), ZSM-48 (d), Z-48-0.1 (e), Z-48-0.2 (f) and Z-48-0.3 (g).

The effect of NaOH treatment on ZSM-22 and ZSM-48 can be directly observed from SEM images (Fig. 3). All ZSM-22 samples exhibit excellent crystallinity with acicular grain morphology. Moreover, the grain length of ZSM-22 decreased slightly along with NaOH treatment concentration increasing. All ZSM-48 samples obtain ellipsoid morphology, and the grain diameter of ZSM-48 decreases from ∼1.0 μm to ∼0.6 μm along with NaOH concentration increasing. Meanwhile, the surface of ZSM-48 grain after NaOH treatment becomes coarser and the shape of particles becomes more irregular. It indicates that NaOH treatment dissolves parts of the surface of zeolites grain, and plays a role of cleaning the surface and reducing the degree of crystalline aggregation. It is in agreement with the trend of the surface area change obtained from N_2_ adsorption/desorption. This result is also in good consistent with the literature reports.^13,14^

**Fig. 4:**
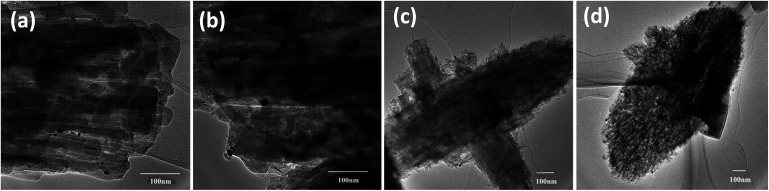
TEM images of Pt/HZ-22 (a), Pt/HZ-22-0.5 mol L^−1^ (b), Pt/HZ-48 (c) and Pt/HZ-48-0.3 (d).

Representative TEM images of Pt supported ZSM-22 (Pt/HZSM-22 and Pt/HZ-22-0.5) and ZSM-48 (Pt/HZSM-48 and Pt/HZ-48-0.3) are shown in Fig. 4. According to the TEM images, mesoporous structures which generated by NaOH treatment can be observed, especially on ZSM-48. All catalysts show a uniform size distribution of Pt particle. Pt particle diameter is centred at 4–10 nm on ZSM-22 and 8–13 nm on ZSM-48. This result suggests nanosized Pt particles are highly dispersed on ZSM-22 and ZSM-48.

**Fig. 5:**
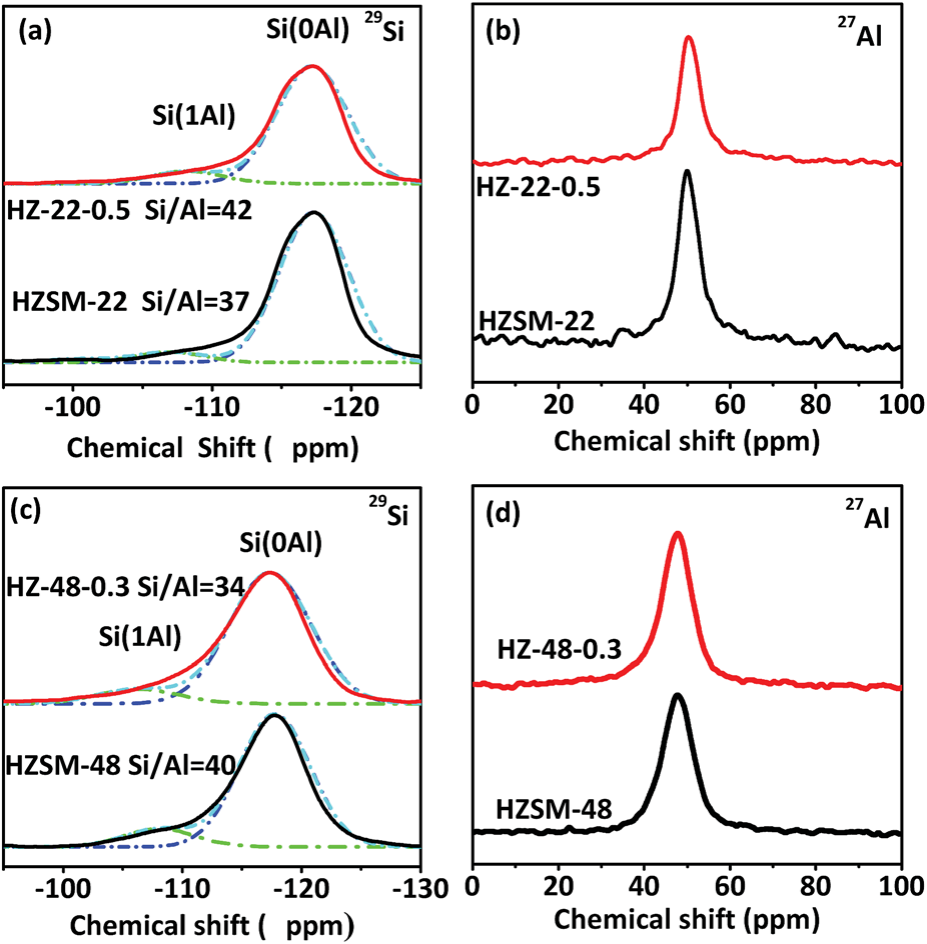
^29^Si and ^27^Al MAS NMR spectra of HZSM-22 (a and b) and HZSM-48 (c and d).

Fig. 5 illustrates ^29^Si and ^27^Al MAS NMR spectra of HZSM-22 (a and b) and HZSM-48 (c and d) samples. In ^29^Si MAS NMR spectra of HZSM-22 and HZSM-48, the intensity of peaks at about *δ* = −117 ppm and *δ* = −107 ppm presenting Si(0Al) and Si(1Al) species, respectively, can be attributed to framework Si. And the intensity of both peaks decreases, indicating that parts of the silica are removed from zeolite framework after NaOH treatment, which have reported in literatures.^15–17^ Meanwhile, as shown in Fig. 5b, the peaks of ^27^Al MAS NMR spectra at *δ* = 49 ppm can be attributed to framework alumina, which slightly decreases after NaOH treatment. This result suggests that the decrease of framework Si and Al on ZSM-22 after NaOH treatment could lead to the decrease of acidity on ZSM-22 after NaOH treatment. Moreover, the decrease of acidity on ZSM-22 after NaOH treatment has been further confirmed by NH_3_-TPD and Py-FTIR. However, as shown in Fig. 5d, the peak of ^27^Al MAS NMR spectra at *δ* = 47 ppm attributed to framework alumina slightly increases after NaOH treatment. The increase of framework Al contributes to the increase of acidity on ZSM-48 after NaOH treatment which has been verified by following acidity investigation. Moreover, the molar ratio of Si/Al has been calculated by ^29^Si MAS NMR spectra. After NaOH treatment, Si/Al of HZSM-22 increases from 37 to 42, then Si/Al of HZSM-48 decrease from 40 to 34. The opposite trend of Si/Al after NaOH treatment suggests NaOH treatment could influence the acidity of HZSM-22 and HZSM-48 diversely.

**Fig. 6:**
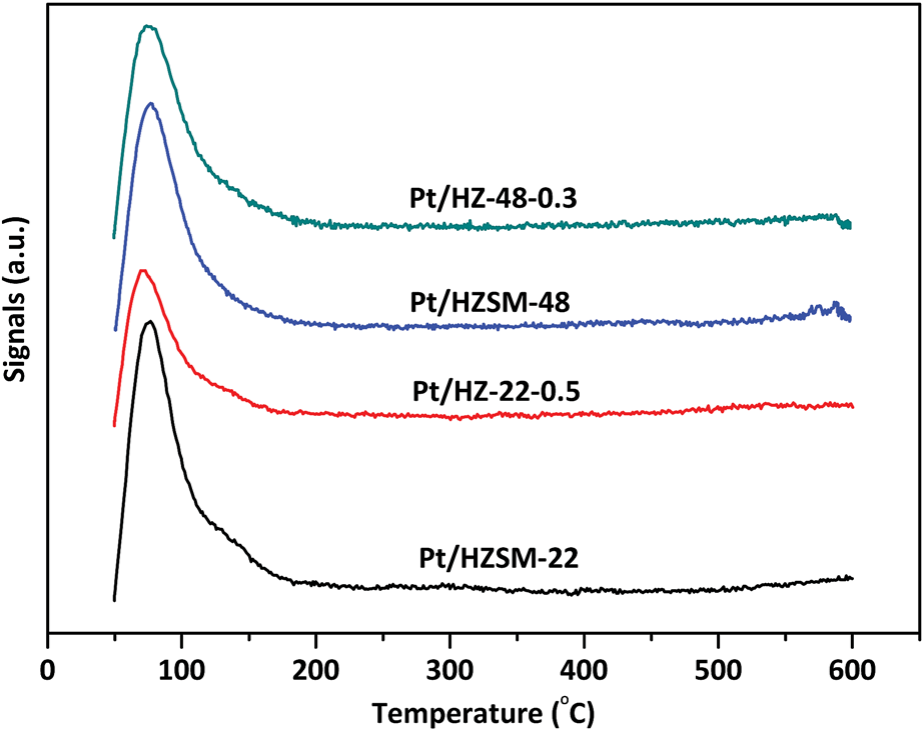
H_2_-TPD spectra of Pt/ZSM-22 and Pt/ZSM-48 samples.

The H_2_-TPD spectra of Pt/HZSM-22 and Pt/HZSM-48 samples are shown in Fig. 6. All samples exhibit a main peaks at ∼80 °C and a shoulder peak a ∼135 °C due to H_2_ desorption. The peak area decreases after alkali treatment on Pt/HZSM-22 samples, and this could be attributed to the relatively large Pt particles sizes of Pt/HZ-22-0.5. This is in accordance with the TEM results. Meanwhile, the peak area of Pt/HZSM-48 decreases slightly after NaOH treatment.

### 3.2 Acidity of ZSM-22 and ZSM-48

**Fig. 7:**
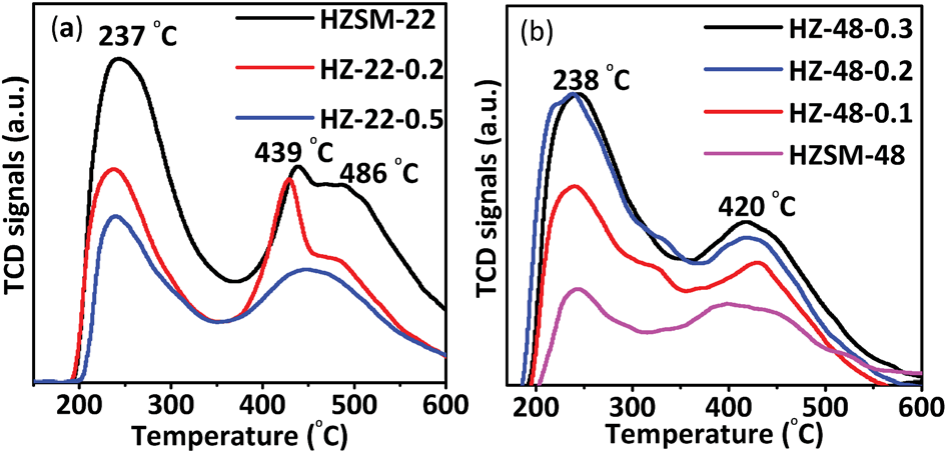
NH_3_-TPD curves of ZSM-22 (a) and ZSM-48 (b) treated with different NaOH concentrations.

Acidity of protonic ZSM-22 and ZSM-48 samples has been tested by both NH_3_-TPD and Py-FTIR. NH_3_-TPD results contain information about density and acid strength of acid sites. NH_3_-TPD curves of ZSM-22 samples are shown in Fig. 7a. All ZSM-22 samples show NH_3_ desorption peaks at ∼237 °C and ∼439 °C corresponding to weak and medium strong acid sites, respectively. In addition, untreated HZSM-22 and 0.2 mol L^−1^ NaOH treated HZ-22-0.2 samples have desorption peak at ∼486 °C corresponding to strong acid sites, while this desorption peak disappears on 0.5 mol L^−1^ NaOH treated sample (HZ-22-0.5). Meanwhile, peak area decreases with resulted in decrease of the acid amount and acid strength on ZSM-22 due to framework silica and alumina removal illustrated by NMR results.

NH_3_-TPD curves of ZSM-48 samples are shown in Fig. 7b. The peaks at ∼238 °C and ∼420 °C corresponding weak and medium acid sites, respectively, can be seen on all ZSM-48 samples. ZSM-48 samples display an opposite trend of acidity by NaOH treatment compared with ZSM-22. Peak area of NH_3_ desorption is gradually increasing with NaOH concentration increasing. This result indicates that the acidity of ZSM-48 has been improved and more acid sites have been generated or exposed by NaOH treatment. The acid amounts of ZSM-22 and ZSM-48 samples are summarized in Table 2. It is known from the literature^9^ that more weak acid sites are beneficial for hydroisomerization, because strong acid sites are prone to adsorb alkanes and produce small molecular hydrocarbons to produce cracking products by side reactions.

**Table 2:** Brönsted and Lewis acid amounts of ZSM-22 and ZSM-48 samples

Sample	*C*_B_ (mmol g^−1^)		*C*_L_ (mmol g^−1^)		*C*_B_/*C*_L_	
	150 °C	300 °C	150 °C	300 °C	150 °C	300 °C
HZSM-22	0.085	0.068	0.018	0.008	4.72	8.5
HZ-22-0.5	0.082	0.066	0.013	0.005	6.31	13.2
HZSM-48	0.046	0.040	0.011	0.008	4.35	4.9
HZ-48-0.1	0.062	0.051	0.032	0.010	1.92	5.09
HZ-48-0.2	0.065	0.049	0.037	0.016	1.73	3.05
HZ-48-0.3	0.092	0.069	0.042	0.023	2.15	2.97

**Fig. 8:**
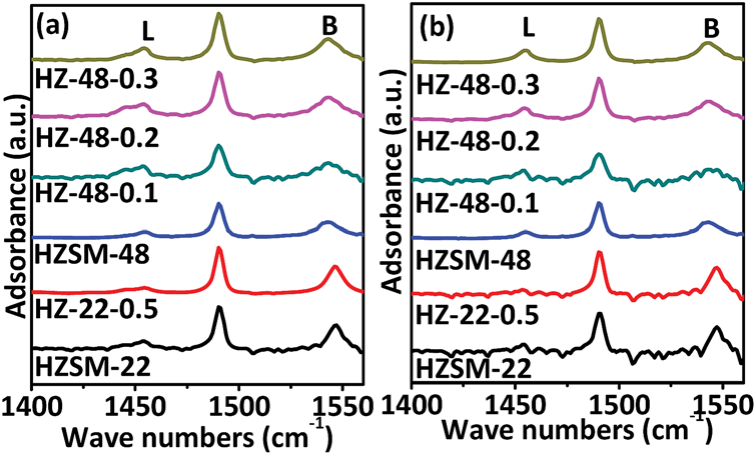
Py-FTIR spectra of ZSM-22 and ZSM-48 samples after pyridine desorption at 150 °C (a) and 300 °C (b).

Acidic properties of ZSM-22 and ZSM-48 samples are also investigated by Py-FTIR (as presented in Fig. 8). Information about the relative changes in the Brönsted and Lewis acid sites can be obtained from Py-FTIR spectra. The bands at 1540 and 1455 cm^−1^ can be assigned to Brönsted and Lewis acid sites, respectively. Pyridine adsorbed peaks 1452 cm^−1^ and 1540 cm^−1^ can be deconvoluted by means of the curve-fitting methods, and then based on the method reported,^18^ Brönsted and Lewis acid sites amounts at different pyridine desorption temperatures are calculated and the results are shown in Table 2.

It can be seen that NaOH treatment process reduces the amount of Brönsted and Lewis acid sites on ZSM-22, while the Lewis acid decreases more. This result is in good consistent with NH_3_-TPD result and the literature report.^19^ Meanwhile, the amounts of Brönsted and Lewis acid sites on ZSM-48 increase gradually with NaOH concentration increasing, and the ratio of Brönsted to Lewis acid amounts (*C*_B_/*C*_L_) decreases with NaOH concentration increasing, indicating. This result is in accordance with the report.^20–22^ According to the variation trend of Brönsted and Lewis acid before and after NaOH treatment of ZSM-22, it can be seen that alkali treatment reduces the total acid amounts of ZSM-22 due to Lewis acids sites decrease, while the Brönsted acid sites almost keep constant. Meanwhile, NaOH treatment process increases the concentration of the Brönsted acid sites on ZSM-48, which should be more beneficial to the isomerization reaction of *n*-alkanes.^23^

### 3.3 Catalytic performances

Catalytic performances of Pt/HZSM-22 and Pt/HZSM-48 samples over *n*-hexane hydroisomerization reaction are investigated. The isomerization products distribution and selectivity for the desired i-C_6_ are discussed at various reaction temperatures.

**Fig. 9:**
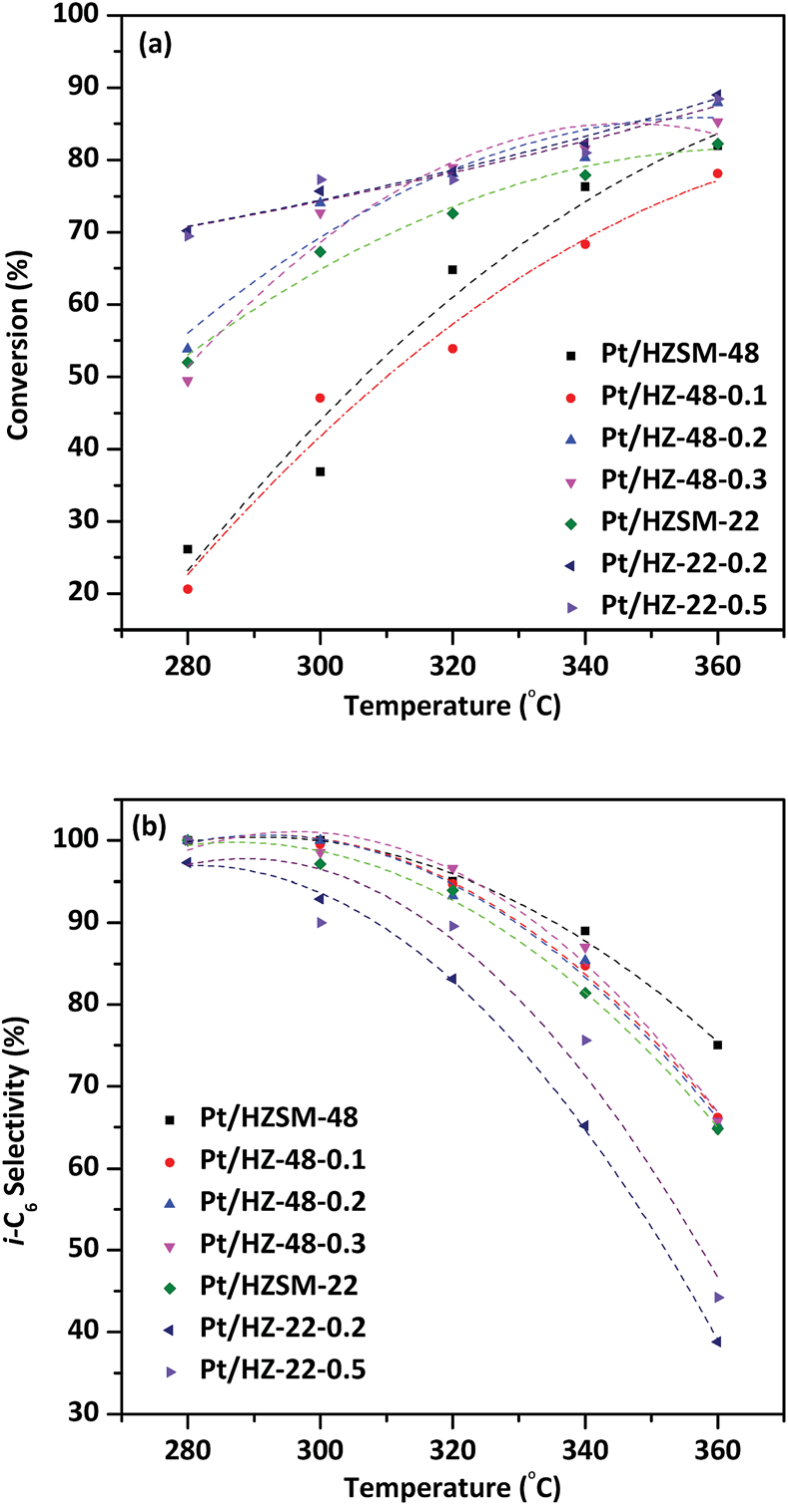
Conversion and i-C_6_ selectivity of *n*-hexane over Pt/HZSM-22 and Pt/HZSM-48 samples.

Fig. 9a shows the effect of temperature on the conversion of *n*-hexane over Pt/HZSM-22 and Pt/HZSM-48 samples. It can be seen from the diagram, the conversion of *n*-hexane for NaOH treatment samples is generally higher than untreated samples. The conversion of *n*-hexane over Pt/HZ-48-0.1 has no obvious change from the untreated sample which is indicated that this concentration of NaOH treatment cannot achieve the effect of significantly changing the catalytic performance. The conversion of *n*-hexane over Pt/HZSM-22 treated with 0.2 mol L^−1^ and 0.5 mol L^−1^ NaOH is about 70%, while the conversion over untreated Pt/HZSM-48 and 0.2 mol L^−1^, 0.3 mol L^−1^ treated concentration is about 50–55% at the temperature is 280 °C. However, conversion over Pt/HZ-22-0.2, Pt/HZ-22-0.5, Pt/HZ-48-0.2 and Pt/HZ-48-0.3 is almost 75% up to 340 °C, while the conversion over untreated Pt/HZSM-22 is 70%. It can be seen from the conversion comparison between Pt/HZSM-22 and Pt/HZSM-48 catalyst that the conversion over Pt/HZSM-22 catalysts is higher than Pt/HZSM-48 catalysts at relatively low temperature (<300 °C). Moreover, the conversion for the two kinds of catalyst is almost the same at higher temperature (360 °C), which reaches close to 90%.

i-C_6_ isomerization product selectivity of the catalysts as a function of temperature is shown in Fig. 9b. The i-C_6_ isomers includes mono-branched isomers (2-MP and 3-MP) and di-branched isomers (2, 2-DMB and 2, 3-DMB). The selectivity to total i-C_6_ decreased with increasing temperature and the selectivity to total i-C_6_ over NaOH treated ZSM-48 catalysts are higher than Pt/HZSM-22 catalysts. At relatively low temperature 280 °C, the two kinds of catalysts obtain almost 100% total i-C_6_ selectivity. However, the total i-C_6_ selectivity of Pt/HZSM-22 decreased sharply to less than 45% and Pt/HZSM-48 decreases to ∼65% at 360 °C. The distinction of selectivity over two kinds of catalysts shows that the Pt/HZSM-48 by NaOH treatment is better in the aspect of hydroisomerization total i-C_6_ selectivity of *n*-hexane than Pt/HZSM-22. The pore structure data obtained by N_2_ adsorption/desorption show that NaOH treated ZSM-48 zeolite obtains larger specific surface area and pore volume than NaOH treated ZSM-22 zeolite, which benefits diffusion of isomerization product on surface and in channel, reducing the retention time to avoid the cracking reaction. Combined with characterizations of the catalysts, the catalytic performances of *n*-hexane hydroisomerization is not only determined by acidity of the catalyst but also influenced by the pore structure of the catalysts.

**Fig. 10:**
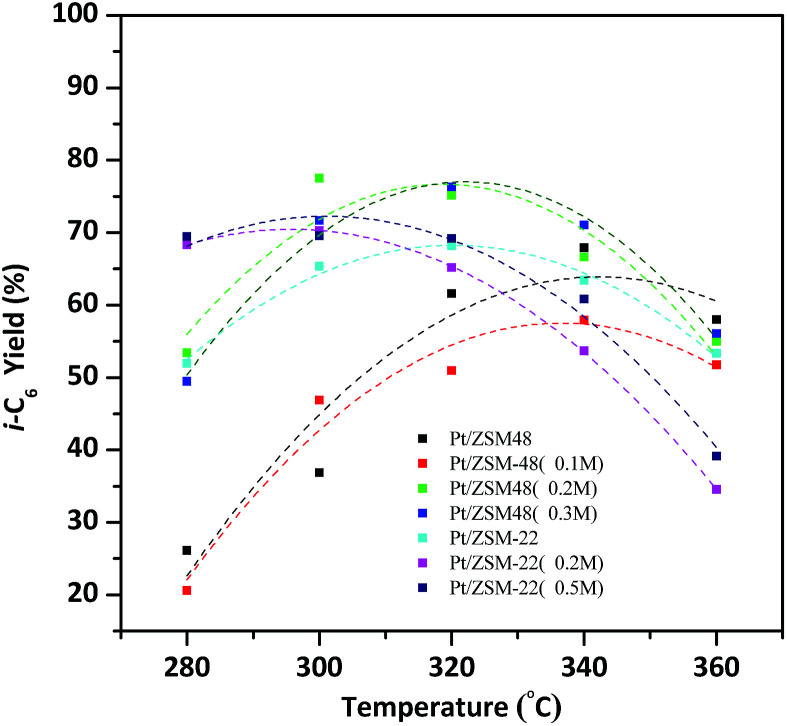
i-C_6_ yield of *n*-hexane over Pt/HZSM-22 and Pt/HZSM-48 samples *versus* temperatures.

On account of isomerization products yield determined by the conversion of reactants and the total i-C_6_ selectivity of products, so the hydroisomerization of *n*-hexane yield over the Pt/HZSM-22 and Pt/HZSM-48 can be more accurately compared. As shown in Fig. 10, by the reason of isomerization products selectivity over the two kinds of catalysts are nearly 100% at 280 °C, then the yield of isomerization products is mainly influenced by the conversion of reactants. The total i-C_6_ yield over Pt/HZSM-22 with NaOH treatment is about 70% and Pt/HZSM-48 with NaOH treatment is between 50–55% at 280 °C. The selectivity of isomerization products over Pt/HZSM-22 with NaOH treatment gradually decreases at temperatures above 300 °C and the conversion *n*-hexane was not quite different from NaOH treated Pt/HZSM-48 catalysts, thus the yield of isomerization products over NaOH treated Pt/HZSM-22 decreases to 70%. However, the yield of isomerization products on NaOH treated Pt/HZSM-48 reaches to 80% at 300 °C, and then decreases due to selectivity of isomers decreasing. Moreover, the i-C_6_ yield is still about 55% at 360 °C on Pt/HZSM-48, while the yield of isomerization product on Pt/ZSM-22 is less than 40%.

The detailed products distribution of *n*-C_6_ hydroisomerization products at 340 °C has been listed in Table 3. The main *n*-hexane hydroisomerization products are mono-branched isomers (including 2-methylpentane (2-MP) and 3-methylpentane (3-MP)) mainly 2-methylpentane (2-MP) on both Pt/HZSM-22 and Pt/HZS-48. Di-branched isomers (2,3-dimethylbutane (2,3-DMB) and 2,2-dimethylbutane (2,2-DMB)) are produced as on all catalysts. Since di-branched isomers obtained higher octane number than mono-branched isomers, alkali treated Pt/HZSM-48 exhibits better catalytic performance compared with Pt/HZSM-22. Over Pt/HZSM-48, the selectivity of di-branched isomers could reach up to 16.3%. Meanwhile, the selectivity of cracking products over Pt/HZSM-48 is lower than Pt/HZSM-22. After NaOH treatment, the acidity of HZSM-22 and HZSM-48 has been modified oppositely, however, the selectivity of cracking products first increases on both catalysts. For Pt/HZMS-22, this could be due to hydrogenation/dehydrogenation ability decreases after alkali treatment which has been confirmed by H_2_-TPD and TEM results. For Pt/HZMS-48, the increase of cracking products selectivity could be due to the acidity increase. However, the selectivity of cracking products decreases after higher NaOH concentration treatment for both catalysts, this can be attributed to the diffusion has been improved after alkali treatment. Pore structure, hydrogenation/dehydrogenation function and acidity affect the hydroisomerization performance at the same time. All three factors should be balanced for better catalytic performance. In general, in our experiments, NaOH treated Pt/HZSM-48 can produce more di-branched isomers and less cracking products than Pt/HZSM-22.

**Table 3:** Products distribution of *n*-C_6_ hydroisomerization products at 340 °C

Samples	Selectivity (mol%)		
	Cracking products	Mono-branched isomers	Di-branched isomers
ZSM-22	18.7	78.9	2.4
Z-22-0.2	34.5	64.5	1.0
Z-22-0.5	23.9	72.5	3.6
ZSM-48	8.4	87.5	4.1
Z-48-0.1	15.2	80.6	4.2
Z-48-0.2	14.6	75.6	9.8
Z-48-0.3	13.0	70.7	16.3

## 4. Conclusions

Alkali treatment will not destroy the structure of ZSM-22 and ZSM-48 at suitable NaOH concentration, while high NaOH concentration will obviously reduce the crystallinity. During alkali treatment, the crystal fragments and amorphous materials on zeolites grains could be cleaned, and the aggregation degree could be reduced. Suitable concentration of NaOH treatment increases the specific surface area and mesoporous volume of ZSM-22 and ZSM-48, meanwhile, the original pore structure have been largely retained. For ZSM-22, the amount of acid sites, especially Lewis acid sites, decreases after NaOH treatment. After NaOH treatment, the amount of Brönsted and Lewis acid sites on ZSM-48 changes in an opposite trend in compared with ZSM-22, which increases after NaOH treated. Conversion, selectivity and yield of *n*-hexane hydroisomerization over NaOH treated Pt/HZSM-22 and Pt/HZSM-48 catalysts are higher than untreated catalysts, indicating that NaOH treatment could improve the efficiency of *n*-hexane isomerization. In addition, the yield of *n*-hexane isomerization products over NaOH treated Pt/HZSM-22 is higher than NaOH treated Pt/HZSM-48 at relatively low temperature (<300 °C), while at higher temperature (>300 °C), NaOH treated Pt/HZSM-48 is superior to NaOH treated Pt/HZSM-22. Meanwhile, after alkali treatment Pt/HZSM-48 could produce more di-branched isomers than Pt/HZSM-22.

## Conflicts of interest

There are no conflicts to declare.

## Supplementary Material

## References

[cit1] Marcilly C. (2003). Present status and future trends in catalysis for refining and petrochemicals. J. Catal..

[cit2] Eswaramoorthi I., Lingappan N. (2003). Hydroisomerization of *n*-hexane over bimetallic bifunctional silicoaluminophosphate based molecular sieves. Appl. Catal., A.

[cit3] Fang K. G., Wei W., Ren J., Sun Y. H. (2004). *n*-Dodecane hydroconversion over Ni/AlMCM-41 catalysts. Catal. Lett..

[cit4] Upadek H., Kottwitz B., Schreck B. (1996). Zeolites and novel silicates as raw materials for detergents. Tenside Surfact. Det..

[cit5] Rahimi N., Karimzadeh R. (2011). Catalytic cracking of hydrocarbons over modified ZSM-5 zeolites to produce light olefins: A review. Appl. Catal., A.

[cit6] Humplik T., Lee J., O'Hern S. C., Fellman B. A., Baig M. A., Hassan S. F., Rahman F., Laoui T., Karnik R., Wang E. N. (2011). Nanostructured materials for water desalination. Nanotechnology.

[cit7] Bellussi G., Pazzuconi G., Perego C., Girotti G., Terzoni G. (1995). Liquid phase alkylation of benzene with 1ight olefins catalyzed by β-zeolites. J. Catal..

[cit8] Groen C. J., Zhu W., Brouwer S., Brouwer S., Huynink S. J., Kaptejin F., Moulijn J. A., Perez-Ramirez J. (2007). Direct demonstration of enhanced diffusion in mesoporousZSM-5 zeolite obtained via controlled desilication. J. Am. Chem. Soc..

[cit9] Gopal S., Smirniotis P. G. (2004). Factors affecting isomer yield for *n*-heptane hydro-isomerization over as-synthesized and dealuminated zeolite catalysts loaded with platinum. J. Catal..

[cit10] Bhattacharya D., Chatterjee M., Sivasanker S. (1997). Studies on the cracking of n-hexane over H-ZSM-48. React. Kinet. Catal. Lett..

[cit11] Wei X., Smirniotis P. G. (2006). Synthesis and characterization of mesoporous ZSM-12 by using carbon particles. Microporous Mesoporous Mater..

[cit12] Su L., Liu L., Zhuang J., Wang H., Li Y., Shen W., Xu Y., Bao X. (2003). Creating mesopores in ZSM-5 zeolite by alkali treatment: a new way to enhance the catalytic performance of methane dehydro-aromatization on Mo/HZSM-5 catalysts. Catal. Lett..

[cit13] Song Y., Zhu X., Song Y., Wang Q., Xu L. (2006). An effective method to enhance the stability on-stream of butane aromatization: Post-treatment of ZSM-5 by alkali solution of sodium hydroxide. Appl. Catal., A.

[cit14] Wang Y., Liu G. Z., Chen C., Wang L. (2012). Influence of Si/Al ratio on catalytic cracking of *n*-dodecane over HZSM-5 membranes. Chem. Ind. Eng..

[cit15] Ogura M., Shinomiya S., Tateno J., Nara Y., Matsukata M., Kikuchi E., Matsukata M. (2001). Alkali-treatment technique-New method for modification of structural and acid-catalytic properties of ZSM-5 zeolites. Appl. Catal., A.

[cit16] Suzuki T., Okuhar T. (2001). Change in pore structure of MFI zeolite by treatment with NaOH aqueous solution. Microporous Mesoporous Mater..

[cit17] Groen J. C., Pérez-Ramírez J., Peffer L. A. (2002). Formation of uniform mesopores in ZSM-5 zeolite upon alkaline post-treatment. Chem. Lett..

[cit18] Emeis C. A. (1993). Determination of integrated molar extinction coefficients for infrared absorption bands of pyridine adsorbed on solid acid catalysts. J. Catal..

[cit19] Danny V., Andre C., Karine T., Jean-Pierre G., Perez-Ramirez J. (2011). Meso-porous ZSM-22 zeolite obtained by desilication: peculiarities associated with crystal morphology and aluminium distribution. CrystEngComm.

[cit20] Zhang M., Li C., Chen Y., Tsang C., Zhang Q., Liang C. (2016). Hydroisomerization of hexadecane over platinum supported on EU-1/ZSM-48 intergrowth zeolite catalysts. Catal. Sci. Technol..

[cit21] Sharon M., Perez-Ramirez J. (2011). Mesoporous zeolites as enzyme carriers: Synthesis, characterization, and application in biocatalysis. Catal. Today.

[cit22] Zhang M., Wang L., Chen Y., Zhang Q., Liang C. (2016). Creating mesoporous in ZSM-48 zeolite by alkali treatment: enhanced catalyst for hydroisomerization of hexadecane. J. Energy Chem..

[cit23] Snehalkumar P., Pant K., Mathew J., Kishore K., Pai S. M., Newalkar B. L. (2015). Hydroisomerization of long chain *n*-Paraffins over Pt/ZSM-22: influence of Si/Al ratio. Energy Fuels.

